# Reinterpretation of oceanic ^230^Th profiles based on decadal export productivity (2003–2010)

**DOI:** 10.1038/s41598-017-00604-y

**Published:** 2017-03-29

**Authors:** Yiming Luo

**Affiliations:** 1 Geophysical Institute, University of Bergen and Bjerknes Centre for Climate Research, Allegaten 70, 5020 Bergen, Norway; 2 Department of Oceanography, Dalhousie University, 1355 Oxford Street, Halifax, NS B3L2H4 Canada

## Abstract

Understanding ^230^Th distributions in the global ocean is important to support and guide the use of this important radioactive nuclide in multiple aspects of oceanographic research, and this issue is now revisited. Based on estimates of export productivity (EP) over 2003–2010 and assuming ^230^Th scavenging at equilibrium at seven Pacific stations where linear ^230^Th profiles are found, an empirical equilibrium-state ^230^Th-scavenging versus EP correlation is established. This correlation demonstrates the relationship between ^230^Th-scavenging intensity and EP in the absence of advection. With this correlation, other ^230^Th profiles from global oceans are reinterpreted. My findings provide alternative explanations of signals carried by ^230^Th distributions with regard to advection, by a reassessment of the ^230^Th deficits in deep waters. Such an equilibrium-state ^230^Th-scavenging versus EP correlation provides a basis for convenient parameterization for use in 3D modeling studies of the ^230^Th distributions.

## Introduction

The naturally occurring uranium-decay series isotopes have been widely used in the oceanographic research. Among all the uranium isotopes and their daughters, ^230^Th (half-life 75.58 kyr), a product by α-decay of ^234^U, is an important one that can be used for multiple purposes^1^. For instance, ^230^Th in sediments can be used as a normalization tool to provide a correction to the estimates of paleo-fluxes^2^ in the ocean, which is important to our understanding of paleoceanographic processes, especially dust inputs from continents^3–5^. Together with ^231^Pa (half-life 32.76 kyr), the daughter of ^235^U, sedimentary ^231^Pa/^230^Th (Pa/Th hereafter) ratios can be used as dynamical proxies to constrain the rate of deep ocean circulation^6^, which plays a key role in regulating global climate^7^. Compared to traditional nutrient-like tracers (e.g., carbon isotopes^8^ (δ^13^C) and Cd/Ca^9^), which only assess the distribution of water masses^6^, this advantage led scientists to successfully apply Pa/Th in a variety of reconstruction of Atlantic Meridional Overturning Circulation (AMOC) for different periods in the past over the last two decades^10–19^.

The apparent success in the application of ^230^Th, despite questions raised about the ^230^Th normalization^20^ or Pa/Th in AMOC reconstruction^21–27^, is based on the simple assumption that ^230^Th produced in the water column is mostly scavenged to the local sediments by settling particles^2, 28^. With a relatively short residence time in the water column (generally 20–40 yr)^1^, ^230^Th is very particle-reactive and its scavenging in sea water was found to be reversible, which is a first order process, best described by the following equation^29^:

1 $${({\rm{Th}} \mbox{-} 230)}_{{\rm{t}}}={{\rm{P}}}_{230{\rm{Th}}}{\rm{Z}}/({\rm{KS}})$$

where (Th-230)_t_ is the total ^230^Th activity at a given depth in the ocean, P_230Th_ is the production rate of ^230^Th in sea water, K is the distribution coefficient of ^230^Th between the particulate phase and the total ^230^Th, S is the average sinking rate of particles, and Z is the water depth.

This simple equation predicts linear profiles of ^230^Th. Linear vertical distributions of ^230^Th are found at many locations in the Pacific Ocean, which are well described by the reversible scavenging model (Equation 1), assuming equilibrium is reached for ^230^Th scavenging^30–33^. However, a recent analysis based on several quasi-linear dissolved water-column ^230^Th profiles in the North Pacific suggests that other parameters, rather than export productivity that is considered to be a typical predictor of scavenging particles, must influence the slopes of water-column ^230^Th profiles^34^.

Moreover, ^230^Th profiles in the global ocean are sometimes curved because ^230^Th distributions can also be influenced by other factors, e.g., advection and bottom scavenging. For example, in the Northwest subtropical Pacific, vertical profiles with ^230^Th-depletion in the deep water are attributed to having enhanced scavenging by particles re-suspended from the sea floor^35^. The curvature of ^230^Th deficits found in the North Atlantic and convex shapes found in the Atlantic sector of Southern Ocean used to be interpreted as the result of overturning circulation^36–41^. Conversely, quasi-linear water-column ^230^Th profiles found in the Equatorial Atlantic and South Atlantic are considered to be the result of water column approaching equilibrium with regard to ^230^Th scavenging^38^, which is also confirmed by a mass balance calculation^42^. However, the outcome of recent Geotraces activities in the North Atlantic shows that ^230^Th profiles are also strongly influenced by particle chemistry, especially in the deep water^43^. This suggests ^230^Th distributions in the ocean are influenced by multiple factors, and even in the Atlantic, where advection is strong, interpretation of ^230^Th signals should be made with caution.

Even if the influence of particle dynamics on ^230^Th profiles is not considered, numerical description of the ^230^Th profiles in the Atlantic as first proposed by Rutgers van der Loeff and Berger^37^, relies on pre-defining the parameters at equilibrium of ^230^Th scavenging. Large uncertainties would thus exist if these equilibrium parameters of ^230^Th scavenging cannot be precisely determined, in interpreting convex profiles with regard to the advection or bottom scavenging effects.

Certainly, multiple factors may influence the ^230^Th distributions in the ocean and there is no consistent view from the community on what factor is dominant. However, a simplified method to analyze ^230^Th profiles with appropriate assumptions is needed, both to test concepts of circulation vs. scavenging from a modeling perspective and to provide an alternative avenue for parameterization in future modeling research for ^230^Th.

In this study, I develop a method to constrain the equilibrium state of ^230^Th scavenging at various locations in the ocean, based on export productivity estimates between years 2003 and 2010 at corresponding ^230^Th-sampling sites. Then I re-examine the ^230^Th distributions at some representative locations based on more appropriate equilibrium state conditions to reinterpret the information we may obtain from the ^230^Th data.

## Results and Discussion

### Equilibrium state of ^230^Th scavenging

In the absence of circulation or mixing, equilibrium state ^230^Th scavenging is defined by Equation 1 and linear vertical profiles of ^230^Th can be found in the water column with slopes depending on the scavenging intensity F_scav_ only (F_scav_ = KS):

2 $${\rm{Z}}/{({\rm{Th}} \mbox{-} 230)}_{{\rm{t}}}={{\rm{F}}}_{{\rm{scav}}}/{{\rm{P}}}_{230{\rm{Th}}}$$

At the same time, linear ^230^Th profiles themselves, if deemed to be in equilibrium, indicate the scavenging intensity changes little with depth throughout the water column (Eq. 2). In the open ocean, where terrestrial particles supplied from rivers or aeolian dusts are very limited, biogenic particles produced in the euphotic zone, as represented by export productivity (EP), become possible dominant scavengers of ^230^Th in the water column. It is therefore reasonable to establish an equilibrium-state ^230^Th-scavenging versus EP, as an alternative to stand for all scavenging particles, for stations where advection effects can be neglected.

**Figure 1:**
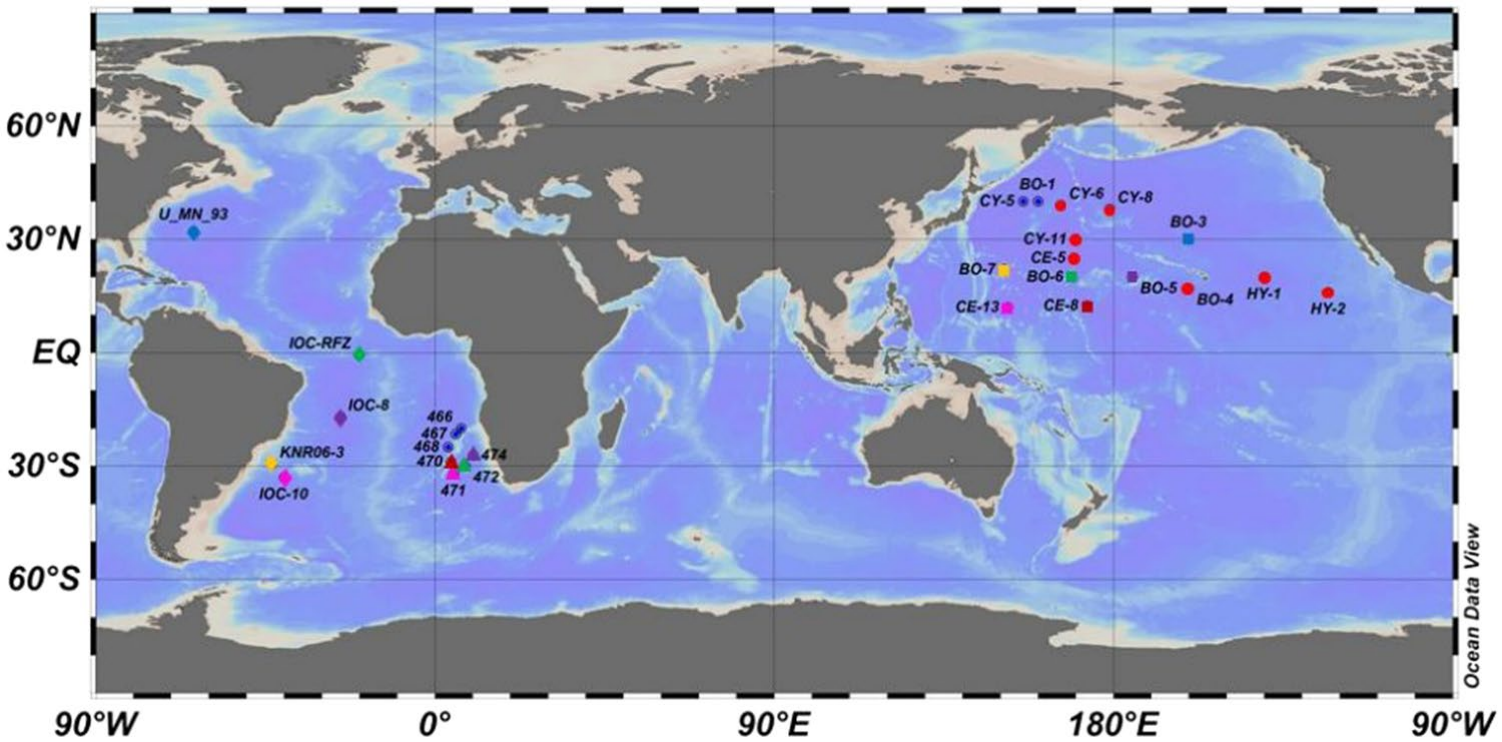
Map for the stations where (^230^Th)_t_ data are available and compiled in this study. Data are retrieved from previous publlications^30, 31, 35, 36, 48, 60–62^. (The profile location colors and symbols correspond to colors and symbols used in Figs 4, 5, 6 and 7.) Figure made with Ocean Data View^63^.

**Figure 2:**
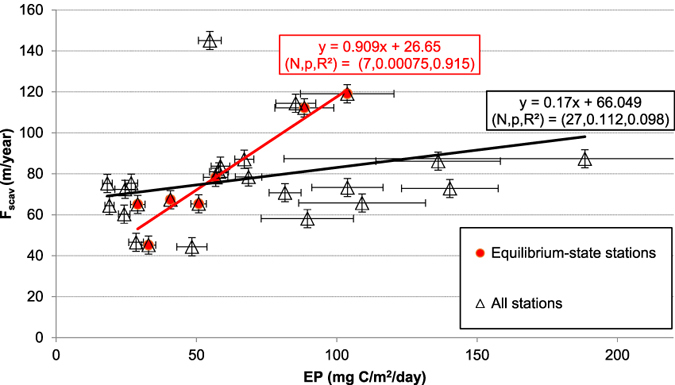
Th-230 scavenging intensity (F_scav_) versus EP derived from the CbPM model output for the stations shown in Fig. 1. The solid black line is the least square linear regression on all the data and the solid red line is the least square linear regression on data from seven Pacific stations, which have the best linearity (R^2^ ≥ 0.96; Table S1).

**Figure 3:**
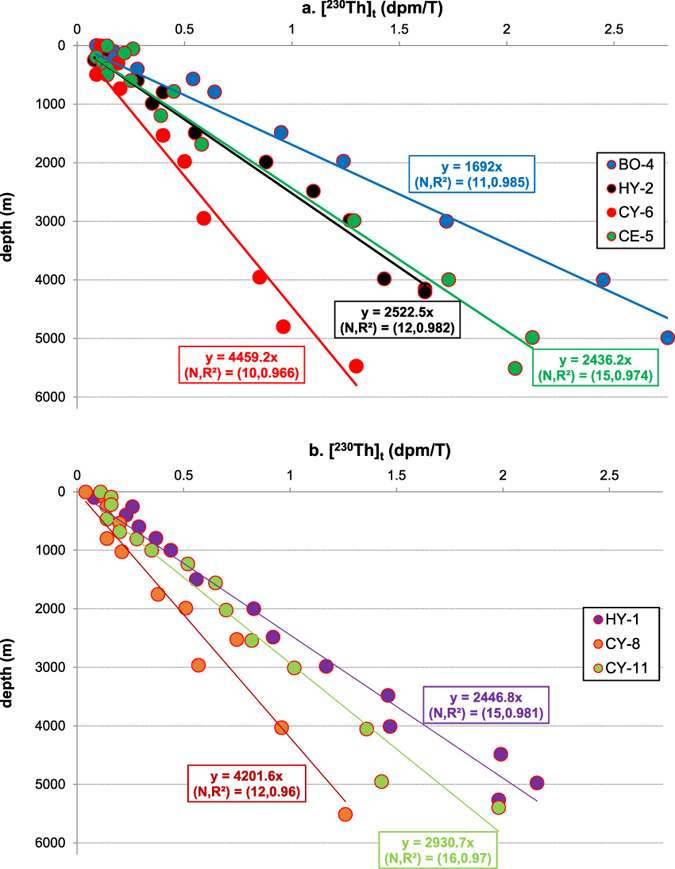
(Th-230)_t_ data from seven Pacific stations and the least square linear regression on the data, which show good linearity of the ^230^Th profiles.

Some previous interpretation of ^230^Th_t_ data was based on comparing the profile to the linear fit of the data, which is presumed as equilibrium-state baseline (e.g., ref. 35). I first plot scavenging intensities of ^230^Th based on the linear fits at all locations compiled for this study against the average EP between years 2003 and 2010, based on NPP from CbPM^44^, for all the stations (Fig. 1; Table S1 in SI; see also Fig. S1 in SI for the productivity variations between profiles); the resulting correlation is weak (R^2^ < 0.1; Fig. 2). Weak correlation suggests that EP does not explain the parameters derived from the linear regression on water column ^230^Th profiles for all the stations. On the other hand, if I choose seven Pacific stations that have the best linear profiles (R^2^ ≥ 0.96; Figs 1, 2 and 3; Table S1) for regression, a very strong least-squares linear fit (R^2^ = 0.916) between the ^230^Th scavenging intensity (F_scav_) and corresponding estimated EP is found.

Near-linear profiles are not inconsistent with some degree of lateral ^230^Th transport^45, 46^, and it is difficult to quantify the lateral transport without more information on the surrounding regional-scale distribution of ^230^Th. Near-linear profiles are however more close to equilibrium-state described by equation 2 as strong lateral processes would likely disturb the linearity of the water column profiles, if the lateral process is not enacted for all depths^46^.

The equilibrium-state ^230^Th-scavenging versus EP correlation is established based on the data from seven Pacific stations, where ^230^Th distributions are assumed to be at equilibrium (Figs 2 and 3),

3 $${{\rm{F}}}_{{\rm{scav}}}=0.9082{\rm{EP}}+26.633$$

where F_scav_ is in m/yr and EP is in mg C/m^2^/day.

This simple correlation provides a basis for our re-interpretation of ^230^Th data at other locations in the world's oceans, in order to test concepts of circulation vs. scavenging at different locations. For stations with an indication of a nonlinear ^230^Th profile, the empirical relationship (3) between F and EP is applied, and the ^230^Th scavenging intensity F_scav_ at those stations is estimated. Then I apply the predicted F_scav_ at those locations to obtain the arbitrary linear ^230^Th profiles for each station, if the advection is absent and only EP, as an alternative for all scavenging particles, is responsible for the ^230^Th distributions at those sites. In subsequent sections, a new approach will be explained and used to re-visit published ^230^Th data in the Atlantic and the Pacific Ocean.

### Th-230 distributions in the West Atlantic along the NADW pathway

**Figure 4:**
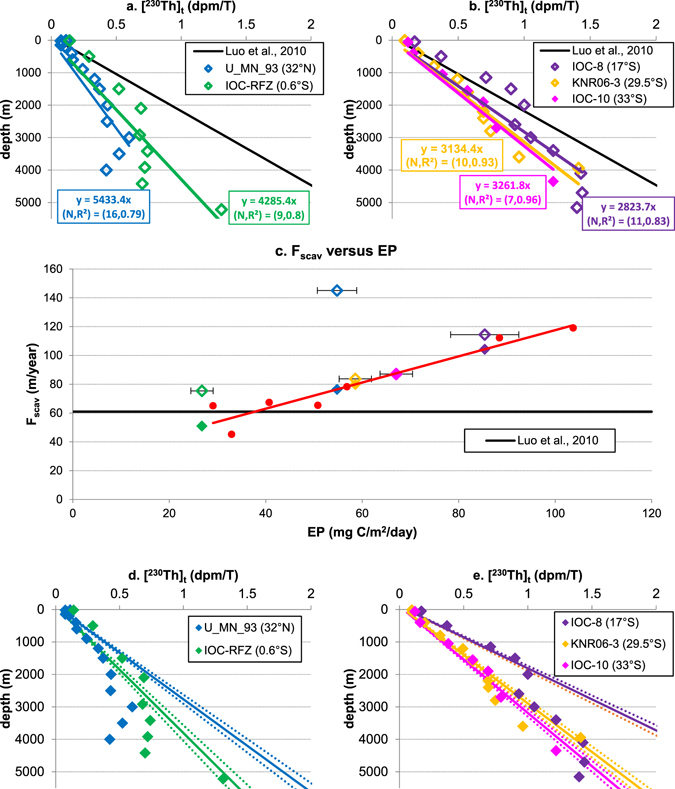
Data analysis for stations in the West Atlantic Ocean. (^230^Th)_t_ data at station U_MN_93, IOC-RFZ (**a**), and station IOC-8, KNR06-3, IOC-10 (**b**) with direct fit of the data using the simple reversible scavenging model (Eq. 1)^29^ and the prediction of (^230^Th)_t_ based on equation 1 using the parameters by Luo *et al*.^36^; (**c**) ^230^Th scavenging intensity at five stations in west Atlantic along the NADW pathway, based on direct fit using equation 1 (colored open diamonds) or determined (colored solid diamonds) by their average local annual export productivity between 2003 and 2010 (EP) and the empirical relationship (Eq. 3) between F_scav_ and EP (red circles are seven stations used to generate the correlation, see Fig. 2); (^230^Th)_t_ data at station U_MN_93, IOC-RFZ (**d**), and station IOC-8, KNR06-3, IOC-10 (**e**) with proposed (solid) linear ^230^Th distributions generated by F_scav_ from (**c**), assuming equilibrium conditions are reached at those locations; dash lines are the uncertainties associated with estimated F_scav_.

The west Atlantic along the NADW pathway is chosen because this is where the NADW influence on ^230^Th distribution is most prominent and thus assessable. Taking published (^230^Th)_t_ data at five stations from North Atlantic to the South Atlantic (Fig. 4; Table S1), there are currently two ways to look at ^230^Th profiles at those locations.

1. One can define a universal equilibrium-state for all the stations and look at the evolution of ^230^Th deficits at depth along the NADW pathway^36, 38^.
  With the universal equilibrium-state setting used by Luo *et al*.^36^ (F_scav_ = 61 m/yr), recovery of the ^230^Th deficit at depth along the NADW pathway from the North Atlantic (station U_MN_93) to the Tropical South Atlantic (station IOC-8) as suggested by François^38^ can be clearly identified (Fig. 4a,b). However, problems emerge when we look at the Tropical to South Atlantic, e.g., this approach indicates ^230^Th deficits increase from 17°S (station IOC-8) to 33°S (station IOC-10) below 2000 m, contrary to the prediction of the North-to-South recovery hypothesis. Assuming a universal equilibrium-state for all the stations is equivalent to assuming an average EP for all the stations. The EP in the ocean is variable and spans over a large range. Therefore, if the actual EP is either too large or too small compared to this ‘average’ number, it will result in underestimation or overestimation of F_scav_.
2. One can also directly fit the data by least squares regression with Equation 1 and compare the real data with the fit (assumed equilibrium at each station).
  A close look at the ^230^Th deficits based on this treatment indicates that ^230^Th deficits in the North Atlantic (station U_MN_93) and Equatorial Atlantic (station IOC-RFZ) are small below about 3000 m (Fig. 4a,b). In South Atlantic, south of 17°S (station IOC-8), ^230^Th scavenging in the water column reaches equilibrium, which is also the conclusion reached by Deng *et al*.^42^.
  This second approach, though apparently reasonable, however suggests ^230^Th-scavenging intensities at some stations that are too high with respect to the EP at corresponding locations if the equilibrium-state ^230^Th-scavenging versus EP correlation suggested in section 2.2 is taken as a reference (Figs 2 and 4c). For example, the fast ^230^Th scavenging rates at stations U_MN_93 (North Atlantic) and IOC-RFZ (equatorial Atlantic) with the low EP are questionable. Moreover, this approach suggests that the measured ^230^Th at depth above 2000 m at station IOC-8 is double that predicted by the EP at that location, which is difficult to explain.
  The above two straightforward ways of interpreting the ^230^Th data appear to fail to provide consistent and reasonable explanations; an alternative approach is therefore needed, which is advanced here:
3. Use the EP estimates for all the stations to back calculate the equilibrium-state scavenging intensity at corresponding stations from Figs 2 and 4c. Then the equilibrium-state linear ^230^Th profiles for all the stations can be plotted and used to calculate the deficits based on the difference between the data and these equilibrium-state profiles (Fig. 4d,e).

In this way, the ^230^Th at 30°N exhibits a large deficit and this deficit gradually decreases along the NADW pathway. The ^230^Th deficit can still be clearly identified at 17°S (station IOC-8) (Fig. 2e), but disappears by 33°S (station IOC-10). This suggests that the water column ^230^Th profiles quickly recover to equilibrium in the Equatorial-South Atlantic regions, as the water mass age of NADW at this location has exceeded the recovery time of ^230^Th with respect to reversible scavenging (Fig. S2 in SI). This evolution of ^230^Th deficits in the deep Atlantic is entirely consistent with the original prediction made by Rutgers van der Loeff and Berger^37^, but it suggests that equilibrium-state for water column ^230^Th is reached in the South Atlantic instead of equatorial Atlantic, as suggested in earlier publications^36, 38^.

Recent studies of dissolved and total ^230^Th^43, 47^ have also demonstrated that enhanced bottom scavenging occurs at some locations. Hayes *et al*.^47^ also discuss how bottom scavenging and circulation effects can be very difficult to disentangle in other locations. My approach based on EP suggests those five total ^230^Th profiles (Fig. 4d,e) show that overturning circulation in the West Atlantic controls the ^230^Th distributions, but it does not rule out bottom scavenging effects. For example, the fast decrease of ^230^Th from 3000 to 4000 m at station U_MN_93 could be explained by bottom scavenging^47^ (Fig. 4d).

### Th-230 distributions in the Cape Basin in the Southeast Atlantic

**Figure 5:**
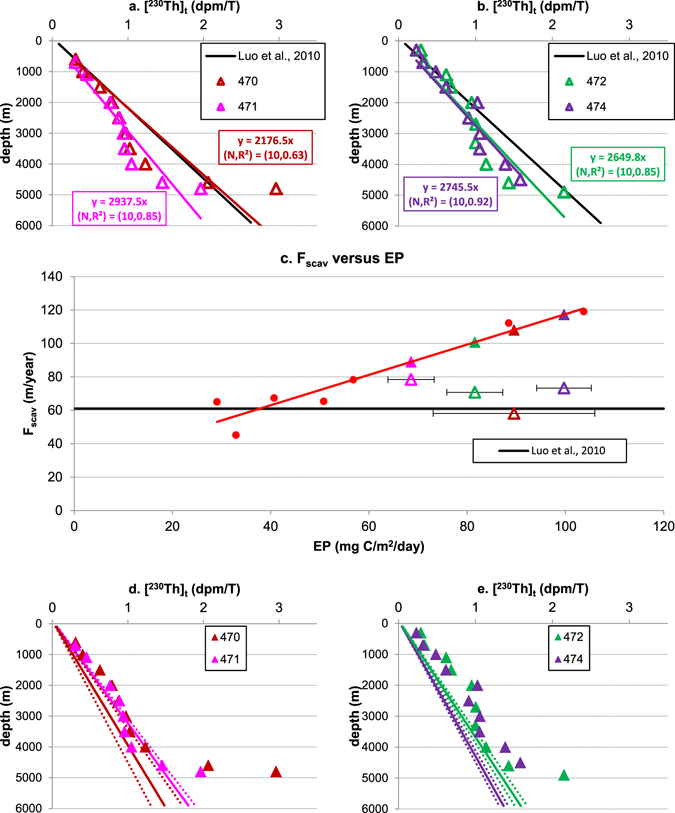
Data analysis for stations in the Southeast Atlantic Ocean. (^230^Th)_t_ data at station 470, 471 (**a**), and station 472, 474 (**b**) with direct fit of the data using the simple reversible scavenging model (Eq. 1)^29^ and the prediction of (^230^Th)_t_ based on equation 1 using the parameters by Luo *et al*.^36^; (**c**) ^230^Th scavenging intensity at four stations in Southeast Atlantic, based on direct fit using equation 1 (colored open triangles) or determined (colored solid triangles) by their average local annual export productivity between 2003 and 2010 (EP) and the empirical relationship (Eq. 3) between F_scav_ and EP (red circles are seven stations used to generate the correlation, see Fig. 2); (^230^Th)_t_ data at station 470, 471 (**d**), and station 472, 474 (**e**) with proposed linear ^230^Th distributions generated by F_scav_ from (**c**), assuming equilibrium conditions are reached at those locations.

Four stations in the Cape Basin in the Southeast Atlantic^48^ (Fig. 5) whose EP falls in the range covered by the calibration stations (Fig. 2; Table S1) are examined next.

By applying the universal steady-state^36^, large ^230^Th deficits at depth below 2000 m at all the stations can be identified (Fig. 5a,b). On the other hand, fitting Equation 1 directly to the data suggests a different interpretation of data. At stations 471 and 472, small ^230^Th deficits are found between 3000 m and 4800 m, and below 4800 m ^230^Th increases rapidly to produce an excess at 5000 m (Fig. 5a,b). Station 470 still features large ^230^Th deficits below 2000 m, while station 474 displays an equilibrium-state linear ^230^Th profiles (Fig. 5a,b).

The above data interpretation indicates ^230^Th deficits at depth in the water column in Cape Basin. The mechanism that would explain the existence of large ^230^Th deficits in the deep water in Cape Basin is, however, difficult to identify. For example, bottom scavenging is not likely to generate these deficits because ^230^Th increases from about 4000 m towards the sea floor.

If the ^230^Th scavenging intensities (F_scav_) derived by directly fitting data are plotted versus EP for all four stations in this region, these values are much lower than the predictions calculated from EP based on the empirical relationship (Eq. 3; Fig. 5c). This suggests F_scav_ values in Fig. 5a and b may be underestimated for high EP. Therefore, by applying the empirical relationship (Eq. 3; Fig. 5c), different F_scav_ values are obtained and suggest an alternative interpretation of ^230^Th data in Cape Basin. The ^230^Th profiles are found to be almost in equilibrium at depth above 4000 m at stations 470, 471 and 472, and they only increase rapidly below 4000 m towards the sea floor. There is excess ^230^Th at 1500–2500 m for stations 472 and 474 that may be controlled by the saline Namib Col Current at depths of about 1300–3000 m at these locations, while the excess of ^230^Th below 3500 m at station 474 can either be a result of expanded AABW that has ^230^Th enriched bottom water, or resuspension of enough ^230^Th-coated sediment in this location (see Fig. 7 in ref. 48).

### Th-230 distributions in the subtropical North Pacific

**Figure 6:**
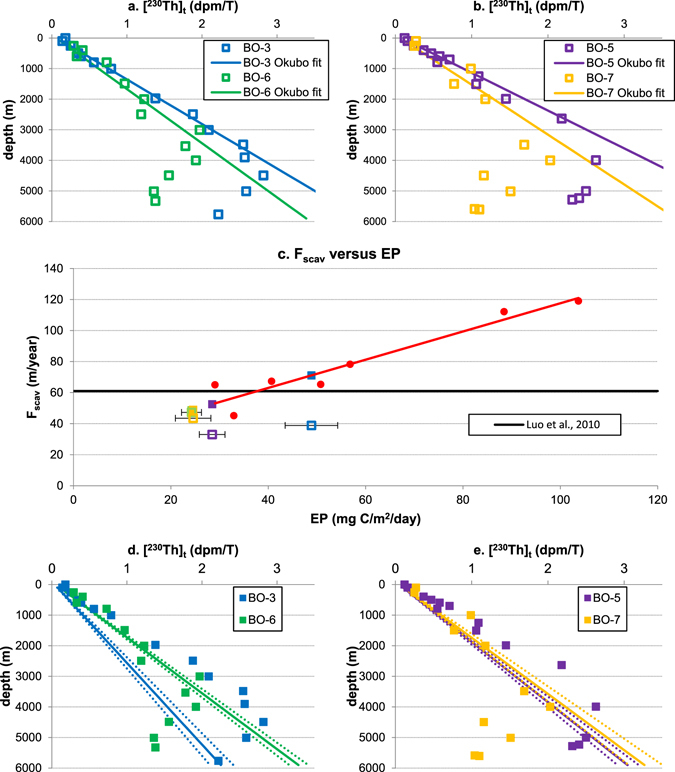
Data analysis for stations in the North Pacific Ocean. (^230^Th)_t_ data at station BO-3, BO-6 (**a**), and station BO-5, BO-7 (**b**) with direct fit used by Okubo *et al*.^35^; (**c**) ^230^Th scavenging intensity (F_scav_) at those stations in North Pacific, based on direct fit using equation 1 (colored open squares) or determined (colored solid squares) by their average local annual export productivity between 2003 and 2010 (EP) and the empirical relationship (Eq. 3) between F_scav_ and EP (red circles are seven stations used to generate the correlation, see Fig. 2); (^230^Th)_t_ data at station BO-3, BO-6 (**d**), and station BO-5, BO-7 (**e**) with proposed linear ^230^Th distributions generated by F_scav_ from (c), assuming equilibrium conditions are reached at those locations.

In the North Pacific, many linear profiles of ^230^Th are observed (Fig. 3), especially in the Northeast Pacific Ocean^30, 31, 35^. However, ^230^Th profiles with curvature shapes are also found there (Fig. 6). By fitting equation 1 directly to the relevant data, Okubo *et al*.^35^ found large ^230^Th deficits below about 3000 m (Fig. 6a,b) at several stations (BO-3, BO-5, BO-6, and BO-7). They argued that bottom scavenging must play an important role in creating those ^230^Th deficits because deep ventilation alone is not sufficient to produce them.

However, the ^230^Th scavenging intensities they used for their fittings are off the empirical relationship (Eq. 3) for stations BO-3 and BO-5 (Fig. 6c), which brings up the question why those stations have such slow ^230^Th scavenging while the local EP is not lower than that at stations BO-6 and BO-7. I re-evaluate the ^230^Th distributions at those stations according to the empirical relationship (Eq. 3) and re-plot the derived linear equilibrium-state ^230^Th profiles in Fig. 6d and e.

At stations BO-6 and BO-7, the F_scav_ values used by Okubo *et al*.^35^ are in line with the predictions from equation 3 (Fig. 6c). This confirms the large deficits below about 3000 m at these two stations (Fig. 6d,e), which can be caused by the joint effects of circulation and bottom scavenging^35^. In fact, stations BO-6 and BO-7 are on the pathway of Lower Circumpolar Water (LCPW)^35^, which was suggested by Fujio and Imasato^49^ to take only a few decades to move from the east of New Zealand to the sub-tropical Northwest Pacific. Modeled deep-water ages at 4500–5000 m from the Norwegian earth system model show that aging of water from the South Pacific east of New Zealand to the sub-tropical Northwest Pacific is about 150 years (Fig. S2 in SI), which is equivalent to the long residence time of ^230^Th at 4500–5000 m in this particular region with respect to removal to the seafloor (SI).

At stations BO-3 and BO-5 however, the F_scav_ values generated by equation 3 are much higher than those used by Okubo *et al*.^35^. As a result, excess ^230^Th over the entire water column at these two stations is predicted (Fig. 5d,e), rather than ^230^Th deficits at depth (Fig. 5a,b). Deep advection is slow at stations BO-3 and BO-5, due to topographic limitations^35^; therefore, deficits caused by ventilation at depth are less likely to exist at these stations due to longer residence time of the deep water^50^. That being said, this reinterpretation of the ^230^Th profiles at these two stations does not conflict with bottom scavenging of ^230^Th. For example, the fast decrease of ^230^Th below 4500 m may still be the result of bottom scavenging^35^.

**Figure 7:**
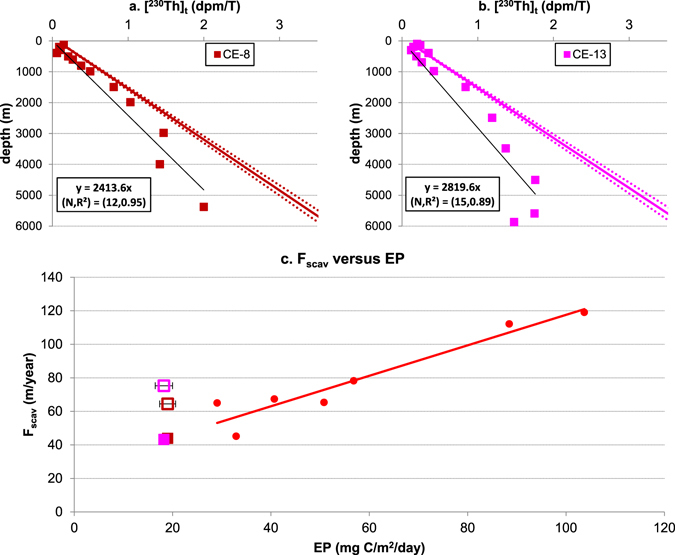
Data analysis for two stations in the West Equatorial Pacific Ocean. (^230^Th)_t_ data at station CE-8 (**a**) and CE-13 (**b**) with direct fit (the black trend lines) of the data using the simple reversible scavenging model (Eq. 1)^29^ and with proposed linear ^230^Th distributions (the colored trend lines) generated by F_scav_ from (**c**), assuming equilibrium conditions are reached at those locations; (**c**) ^230^Th scavenging intensity at two stations in West Equatorial Pacific Ocean, based on direct fit using equation 1 (colored open squares) or determined (colored solid squares) by their average local annual export productivity between 2003 and 2010 (EP) and the empirical relationship (Eq. 3) between F_scav_ and EP (red circles are seven stations used to generate the correlation, see Fig. 2).

Station CE-8 and CE-13, upstream of BO-6 and BO-7 on the route of LCPW^35^, provide another case for us to examine with equation 3. If the ^230^Th deficits at depth below about 3000 m are partly caused by ventilation of LCPW at BO-6 and BO-7, as suggested by Okubo *et al*.^35^, the same or even stronger deficits should be expected at CE-8 and CE-13. However, these deficits can not be elucidated by fitting linear regressions to the data (Fig. 7a,b). The EP at CE-8 and CE-13 are low and outside the range of the EP of the calibration stations. When the empirical relationship (Eq. 3) is applied and F_scav_ values at these sites are calculated (Fig. 7c), the results suggest a deficit in ^230^Th at all depths at those locations (Fig. 7a,b).

### Implications

I have addressed issues associated with applying a simple universal equilibrium state scavenging in studying the ^230^Th distributions in the world's oceans, as done by Luo *et al*.^36^. When measured local EP, where water column ^230^Th data are available, is either too high (e.g., >60 mg C/m^2^/day) or too low (e.g., <30 mg C/m^2^/day), it will result in a ^230^Th scavenging intensity that deviates from the presumed value used by Luo *et al*.^36^ (Figs 4c, 5c, 6c and 7c). Applying the empirical relationship (Eq. 3; Fig. 2) in order to better constrain the scavenging intensities at various locations in the ocean is thus highly recommended for future research especially in modeling. However allocating accurate scavenging parameters for each water column location (depending on the horizontal resolution of a specific model) can be labor-intensive and requires a larger dataset over a long time interval, especially for high-resolution 3D models.

**Figure 8:**
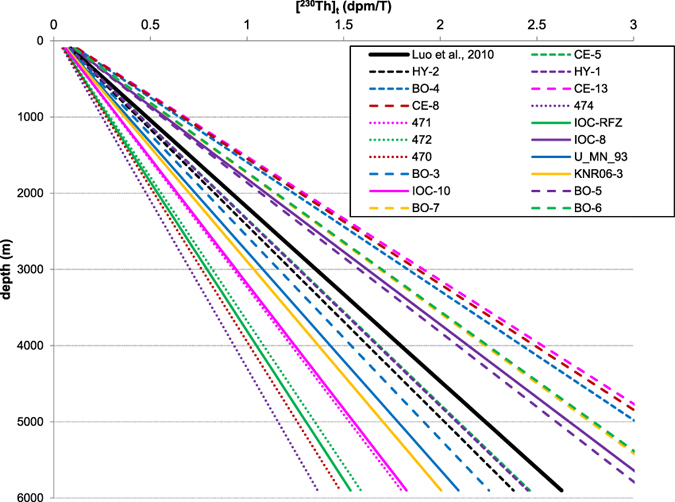
Equilibrium-state linear ^230^Th profiles for all the stations, dictated from the empirical F_scav_ versus EP correlation (Fig. 2) and local average EP over 2003–2010. Solid black lines are the universal steady-state profiles used by Luo *et al*.^36^.

On the other hand, applying a universal equilibrium state for ^230^Th scavenging can capture signals of advection from ^230^Th records in the Atlantic (Fig. 4a,b). When suggested equilibrium-state linear ^230^Th profiles at all stations were plotted together with the universal equilibrium state of ^230^Th scavenging used^36^, F_scav_ values employed by Luo *et al*.^36^ fell in the range derived from all the stations (Fig. 8).

This suggests the parameters used by Luo *et al*.^36^ may represent the global average F_scav_ for ^230^Th, and suggests that these should still be useful in studying the basin-wide ^230^Th distribution patterns. Extreme cases, such as rapid ^230^Th scavenging at high EP region (e.g., 470, 474 and IOC-RFZ) or slow ^230^Th scavenging at low EP region (e.g., CE-13, CE-8 and BO-4), may however complicate the data interpretation and need to be treated carefully.

### Uncertainties and limitations

Although I have shown that the new relationship between CbPM derived EP and ^230^Th scavenging rates can be used to study ^230^Th distributions in the ocean, there are still some uncertainties and limitations that need to be addressed.

The first issue is the uncertainties with regard to EP (export of particulate organic carbon, POC, from the euphotic zone) estimation. The only method to retrieve the long-term record of net primary production in the ocean is based on models that use satellite remote sensing products and whose output is tested by primary production rates inferred from C-14. Beyond the uncertainties associated with those remote sensing products, different models^44, 51^ (e.g., CbPM and VGPM) generate different results in certain regions. Those uncertainties are difficult to document and they are further complicated by uncertainties associated with the estimation of the depth of the euphotic zone^52^ (Z_eu_) and the approach in calculating EP^53^ from NPP, SST and Z_eu_. In fact, during the process of analyzing those model products, I found that NPP for mid-low latitude regions between 45°N and 45°S is more stable, while NPP for high latitude regions is highly variable, with some winter time predictions even reaching zero^44^. The process of calculating EP has similar problems, and EP becomes greatly variable at high latitude when the SST, NPP and Z_eu_ have large seasonal variability. Therefore, it is probably more reliable to use the empirical relationship (Eq. 3) only for mid-low latitude regions between 45°N and 45°S in future research.

Besides the uncertainties with regard to EP, the rationale for using EP as a single predictor to stand for all particles that are related to ^230^Th scavenging in the water column (F_scav_ = KS) is based on the assumption that particles scavenge ^230^Th at constant rate and all the particles’ abundance co-varies with EP. Recent studies have shown that thorium has higher affinity to organic matter, as compared to protactinium, and thorium is not as sensitive as protactinium to particle composition during adsorption on particles in seawater^54, 55^. However, the differences in adsorbing Thorium on different particles are still evident^54, 56^. Furthermore, the composition of particles exported from the ocean’s euphotic zone shows large regional variations, which means different components may not co-vary with EP. These issues bring some uncertainties that are difficult to constrain.

Lastly, spatial coverage of ^230^Th_t_ is sparse and the determination of empirical correlation between EP and F_sacv_ is based on stations whose EP are in a range between about 29 and 104 mg C/(m^2^day). Therefore, it would be the best to apply the empirical relationship (Eq. 3) in this EP range. EP calculated for ocean regions between 45°N and 45°S is generally above 15 mg C/(m^2^day) and most falls between 29 and 104 mg C/(m^2^day), excluding some marginal and upwelling areas. Therefore, the empirical relationship (Eq. 3) can surely be applied for most ocean regions between 45°N and 45°S.

In conclusion, I systematically re-evaluate some of the representative ^230^Th data from both Atlantic and Pacific with an empirical relationship (Eq. 3; Fig. 2) derived by examining ^230^Th profiles and corresponding average EP between years 2003 and 2010 at seven stations which are considered to be at equilibrium with regard to ^230^Th scavenging. My approach provides an alternative way to interpret the ^230^Th distributions in the ocean, and the established empirical equilibrium-state ^230^Th-scavenging versus EP correlation (Eq. 3) provides a basis for better parameterization for use in 3D modeling studies of ^230^Th distributions. Future research in this field will have to centre on improving the computing algorithm for primary productivity, in order to make the method applicable to a broader region in the ocean. More ^230^Th data are also highly desired to both further support the method and provide more cases to analyze with the method.

## Methods

### Justification of using remote sensing products

As a dominant source for settling particles in the open ocean, export productivity (EP) plays a key role in ^230^Th scavenging in the water column^57^, and it is used as an alternative for all scavenging particles. The most accurate approach to derive observational estimates of EP is direct measurements of primary production, based on C-14 incubation rates. However, primary production rates inferred from C-14 are scarce and do not cover long time periods, as do satellites. Limited EP measurements, and especially the lack of long term records at the sites where water column ^230^Th data are available, make evaluation of the effect of EP on ^230^Th scavenging difficult.

Here, I choose some of the most well-developed algorithms (e.g., ref. 44) to calculate primary productivity, because models based on remote sensing products is the only practical way to measure global ocean primary productivity, though inconsistencies between satellite-based and *in-situ* primary productivity data do exist^58^. Then I take a standard approach to estimate long-term EP at chosen sites, using satellites data and relevant computing algorithms for net primary productivity^44, 51^, euphotic depth^52^ and export ratios^53^ (ef) between January, 2003 and December, 2010. I take the monthly averaged EP and use these results to study the ^230^Th-scavenging in the ocean.

### Data preparation for EP

The Moderate Resolution Imaging Spectroradiometers (MODIS) aboard satellites provide data on surface chlorophyll concentrations (Chl), sea surface temperature data (SST), and cloud-corrected incident daily photosynthetically active radiation (PAR). Those data were used by the Carbon-based Productivity Model (CbPM^44^) and the Vertically Generalized Production Model (VGPM^51^) to estimate monthly-averaged ocean net primary production in 1/6° × 1/6° resolution. Export production was then calculated between January 2003 and December 2010 based on the net primary production, SST, and euphotic depths^52^ using the method from Laws *et al*.^53^ (See Supplementary Information for details of these calculation).

I take the average EP over the period between 2003 and 2010 because ^230^Th has a long residence time in the water column. (Note that no remote sensing products were available before 2002 for net primary production (NPP) calculation) The residence time of ^230^Th increases with depth in the water column generally to 20–40 years in the deep ocean^1^. Thus, ideally the EP during 40 years before the sampling date would need to be taken into consideration. On the other hand, most of the EP estimates over 2003–2010 show small annual deviation of 5–15% (Table S1), so that the average EP values over 2003–2010 should be representative of the long-term (40 years) situation at different sites, with a limitation of this approach by assuming no significant ecological changes have occurred over the past few decades.

It is worth mentioning that EP estimated using NPP from CbPM^44^ and that from VGPM^51^ are close (Table S1), though the two models do generate different NPP output, particularly in the subtropics (http://www.science.oregonstate.edu/ocean.productivity/custom.php). The reason could be either the chosen sites in this study do not fall in the region that predictions of the two models diverge, or the approach to calculate EP from NPP reduces the difference between the two NPP outputs as the same SST and euphotic depths^52^ are used. As a result, the correlations between F_scav_ and EP generated using two models are close (Fig. S1). Considering this consistency in calculated EP and that CbPM may provide a more accurate estimate of NPP, rather than the chlorophyll-based VGPM^59^, only EP calculated from NPP generated by CbPM is reported in the main text.

### Data preparation for ^230^Th

Published water column total ^230^Th data^30, 31, 35, 36, 48, 60–62^ at 27 locations where water depth is greater than 3500 m and with more than 7 data points were compiled (Fig. 1 and Table S1 in SI). Dissolved ^230^Th is more frequently sampled, especially in the ongoing GEOTRACES program, and dissolved ^230^Th generally makes 85–90% of total ^230^Th. Dissolved ^230^Th data are however excluded from this study because the influence of the particulate fraction on the slope of the ^230^Th profiles (dissolved versus total) can still be very significant.

## Electronic supplementary material

Supplementary Information
