# Supplementary material for: Reinterpretation of oceanic 230Th profiles based on decadal export productivity (2003–2010)
Source: Sci Rep. 2017 Mar 29;7:505. doi: 10.1038/s41598-017-00604-y (PMC5428837; doi:10.1038/s41598-017-00604-y)
Supplement: Supplementary file 1 — Supplementary Information [file 41598_2017_604_MOESM1_ESM.doc]

Supplementary Information for

**Reinterpretation of oceanic ^230^Th profiles based on decadal export productivity (2003-2010)**

Yiming Luo

University of Bergen

**Contents of this file**

Figures S1-S3, Tables S1

**Introduction**

The primary productivity (PP) estimates are based on CBPM and VGPM model, which can be retrieved on this website: <http://www.science.oregonstate.edu/ocean.productivity/>. The PP data used in this study are derived from the MODIS datasets, and relevant MODIS data can also be found on the same web. Then I used the method developed by Laws et al. (2000) to calculate the export productivity (EP). The ^230^Th_total_ data are compiled from earlier publications (Nozaki et al., 1981; 1987; Moran et al., 2001; 2002; Scholten et al., 2008; Luo et al., 2010; Anderson et al., 2012; Okubo et al., 2012).

In order to allocate correct EP value to the right station where ^230^Th is available, the [coordinates](http://en.bab.la/dictionary/english-chinese/coordinates) of the stations are used to determine the corresponding grid-box in the MODIS data file, according to the resolution of the MODIS data.

The MatLab scripts used to generate the EP results are available from the corresponding author. The code is not necessarily user-friendly, but its key sections are documented.


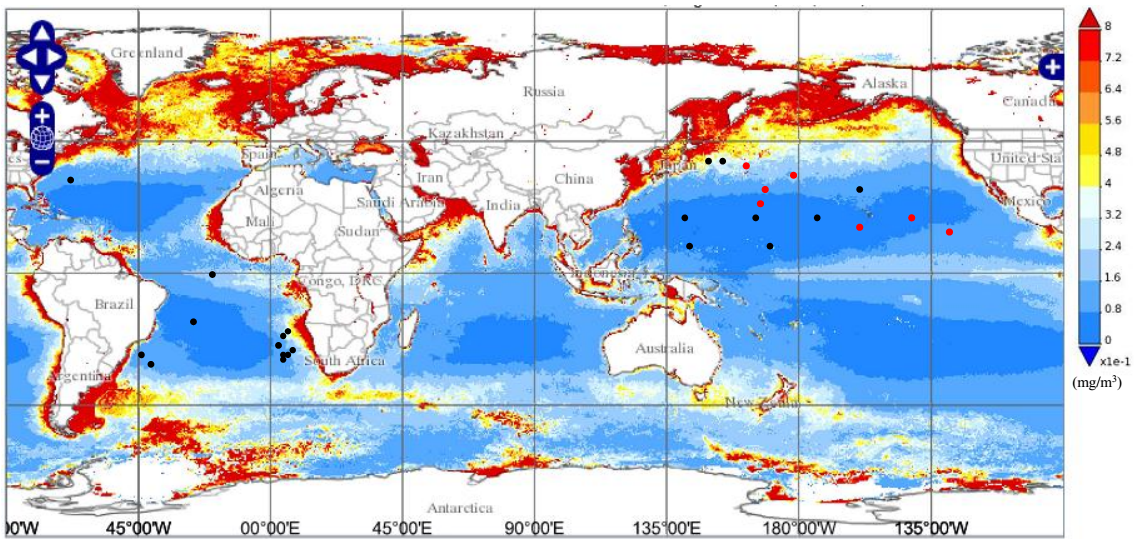


**Figure S1.** Map of Monthly averaged Chlorophyll a concentration based on MODIS data between January 2003 and December 2003. Circle symbols correspond to the stations where (^230^Th)_t_ data are compiled in this study (Table S1), as shown in Fig. 1. This figure is generated using the Giovanni plotting service (http://giovanni.sci.gsfc.nasa.gov/giovanni/).


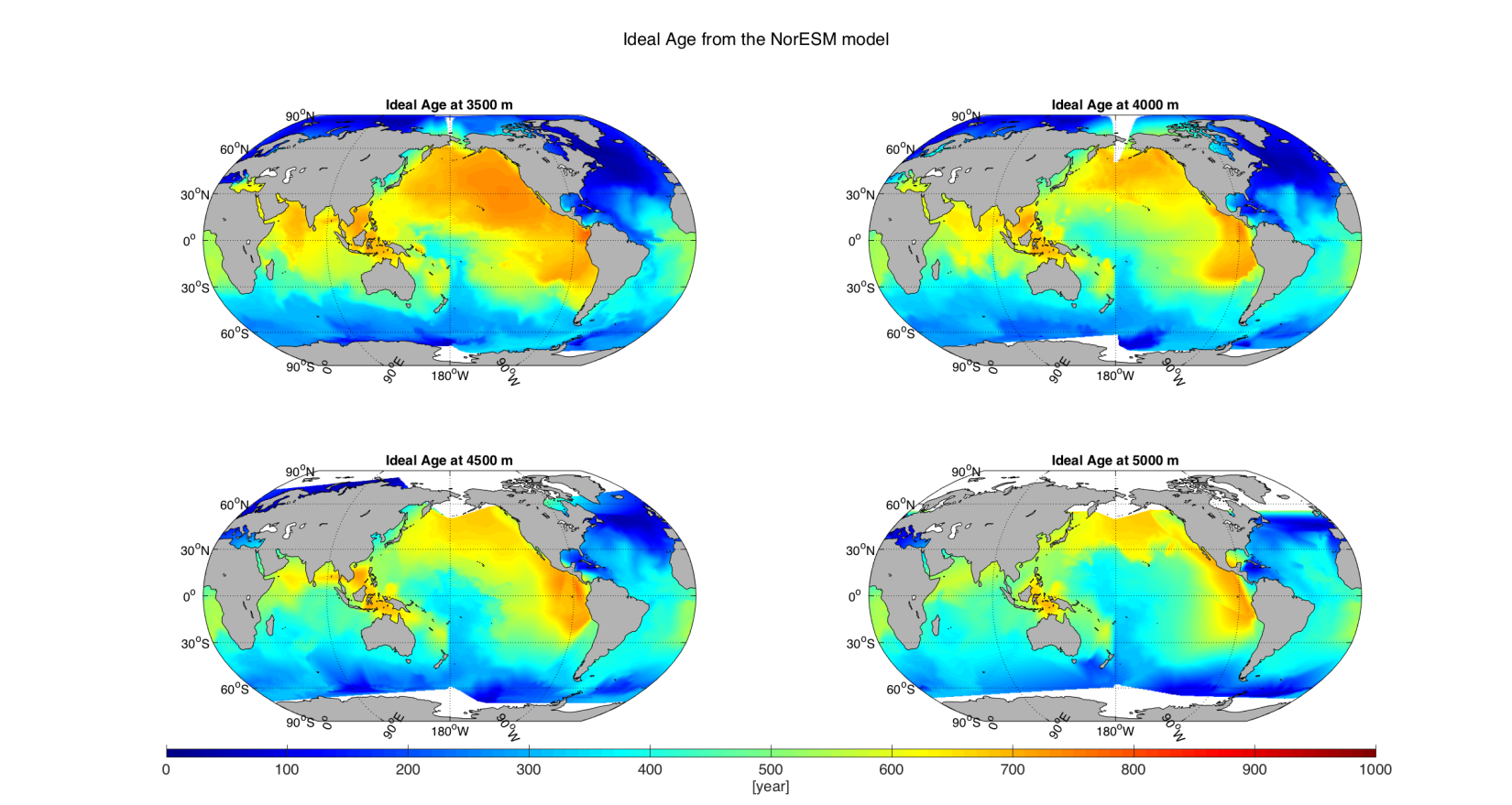


**Figure S2**. Modeled deep water mass ideal age from the Norwegian Earth System Model (NorESM: <https://verc.enes.org/models/earthsystem-models/ncc/noresm>). The figure is created by using M_map function (<https://www.eoas.ubc.ca/~rich/map.html>) in MATLAB (version 2015b) .


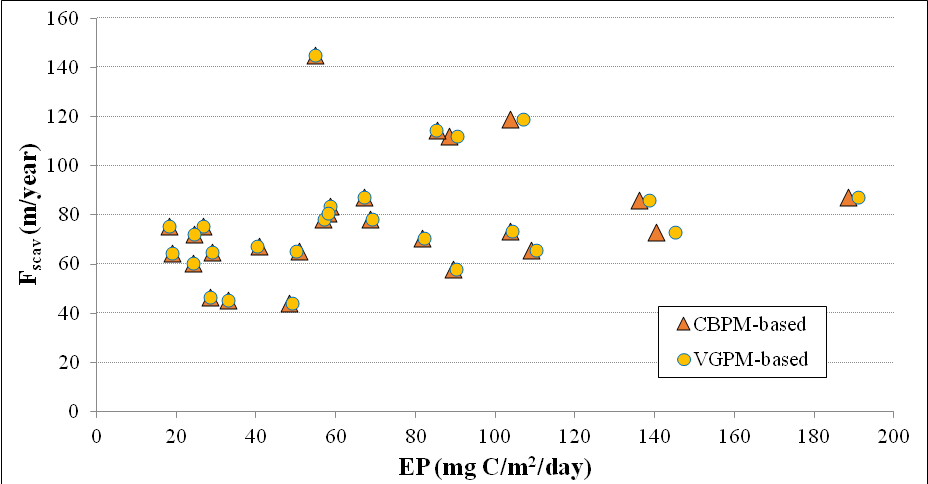


**Figure S3.** F_scav_ versus EP estimates based on both CBPM and VGPM PP

**Table S1:** Locations of ocean stations where ^230^Th_total_ data are compiled, average Export Productivity between 2003 and 2010 based on Primary Productivity from CBPM and VGPM with annual deviation, and the fitting parameters when I apply linear regression on the ^230^Th data at those locations.

| station | latitude | longitude | location | EP- CBPM | Annual | EP-VGPM | Annual | Linear Fit R^2^ | F_scav  by fitting |
| --- | --- | --- | --- | --- | --- | --- | --- | --- | --- |
|  |  |  |  |  | deviation |  | deviation |  |  |
| BO-4 | 17 | -160 | NE Pac | 32.93 | 2.57 | 32.9 | 2.57 | 0.98 | 45.1764 |
| HY-2 | 16 | -123 | NE Pac | 40.7 | 1.36 | 40.28 | 1.59 | 0.98 | 67.35075 |
| HY-1 | 20 | -140 | NE Pac | 50.79 | 2.78 | 49.92 | 2.98 | 0.98 | 65.32956 |
| CE-5 | 25 | 169.6 | N Pac | 29.07 | 2.56 | 28.92 | 2.62 | 0.97 | 65.04654 |
| CY-11 | 30 | 170 | N Pac | 56.82 | 4.32 | 56.98 | 4.1 | 0.97 | 78.24969 |
| CY-6 | 39 | 166 | N Pac | 103.72 | 16.67 | 106.99 | 16.78 | 0.97 | 119.06064 |
| CY-8 | 38 | 179 | N Pac | 88.45 | 10.51 | 90.38 | 11.85 | 0.96 | 112.18272 |
| IOC-10 | -33 | -40 | SW Atl | 67.04 | 3.38 | 67.16 | 3.35 | 0.96 | 87.09006 |
| CE-8 | 12.45 | 173 | EW Pac | **18.99** | 1.62 | 18.85 | 1.56 | 0.95 | 64.44312 |
| BO-1 | 40 | 160 | NW Pac | **140.29** | 17.24 | 145.07 | 21.11 | 0.93 | 72.84027 |
| KNR06-3 | -29.5 | -43.3 | SW Atl | 58.54 | 3.35 | 58.52 | 3.32 | 0.93 | 83.68848 |
| 474 | -27.2 | 10.15 | SE Atl | 103.81 | 12.7 | 104.16 | 12.76 | 0.92 | 73.30485 |
| CY-5 | 40 | 156 | NW Pac | **136.13** | 22.18 | 138.47 | 23 | 0.92 | 86.16624 |
| 467 | -21.5 | 5.5 | SE Atl | **109.01** | 22.59 | 110.16 | 23.71 | 0.89 | 65.74608 |
| CE-13 | 12 | 152 | EW Pac | **18.23** | 1.75 | 18.07 | 1.62 | 0.89 | 75.28332 |
| BO-5 | 20 | -175 | N Pac | 28.48 | 2.63 | 28.42 | 2.62 | 0.89 | 46.54077 |
| BO-3 | 30 | -160 | N Pac | 48.37 | 5.39 | 49 | 5.49 | 0.88 | 44.34336 |
| 472 | -30 | 8 | SE Atl | 81.55 | 5.69 | 82.03 | 5.83 | 0.85 | 70.74966 |
| 471 | -32 | 5 | SE Atl | 68.57 | 4.7 | 69 | 4.7 | 0.85 | 78.43125 |
| IOC-8 | -17 | -25 | EW Atl | 26.75 | 2.31 | 26.73 | 2.28 | 0.83 | 75.39279 |
| 466 | -20 | 7 | SE Atl | **188.39** | 107.21 | 191.1 | 110.66 | 0.8 | 87.34638 |
| IOC-RFZ | -0.59 | -20.03 | EW Atl | 85.38 | 7.08 | 85.24 | 7.14 | 0.8 | 114.42018 |
| U_MN_93 | 32 | -64 | NW Atl | 54.76 | 4.09 | 54.79 | 4.07 | 0.79 | 145.07178 |
| 468 | -25 | 3.5 | SE Atl | 58.02 | 3.34 | 58.18 | 3.48 | 0.76 | 80.96508 |
| BO-6 | 20 | 169 | NW Pac | **24.24** | 2.06 | 24.18 | 2.08 | 0.63 | 60.28593 |
| 470 | -29.85 | 4.3 | SE Atl | 89.53 | 16.45 | 90.28 | 17.07 | 0.63 | 58.11255 |
| BO-7 | 22 | 151 | NW Pac | **24.57** | 3.65 | 24.54 | 3.64 | 0.41 | 72.41307 |
